# CaMKII in Cardiometabolic Disease

**DOI:** 10.18632/aging.100675

**Published:** 2014-06-22

**Authors:** Lale Ozcan, Ira Tabas

**Affiliations:** ^1^ Department of Medicine, Columbia University, New York, NY 10032; ^2^ Department of Pathology & Cell Biology, Columbia University, New York, NY 10032; ^3^ Department of Physiology & Cellular Biophysics, Columbia University, New York, NY 10032

The worldwide epidemic of obesity is closely linked to insulin resistance and type 2 diabetes (T2D), which have led to a critical need for new drug development. Insulin resistance and T2D contribute to the pathogenesis of many diseases including fatty liver disease, cardiovascular disease, kidney failure and retinal disease. Moreover, the incidence of T2D increases with age, and several epidemiological studies have shown that it is directly related to vascular dementia and Alzheimer disease in the elderly population (Samaras et al. Ther. Adv. Endocrinol. Metab., 2012, 3:189-96). A growing body of work indicates that increased hepatic glucose production is central to the pathophysiology of these alterations. Excessive glucagon signaling, due to elevated glucagon levels and lack of glucagon suppression, in the setting of insufficient hepatic insulin action contribute to disproportionate hepatic glucose production (Lin and Accili Cell Metab., 2011, 14:9-19).

Recent work from our lab has shown that excessive glucagon action in obesity, through protein kinase A-mediated phosphorylation and activation of the inositol 1,4,5-trisphosphate receptor (IP3R) ER calcium channel, promotes excessive calcium release into the cytoplasm (Wang et al. Nature, 2012, 485:128-32). This process is exacerbated by defective cytoplasm-to-ER transport of calcium owing to lipid-mediated sarco(endo)plasmic reticulum Ca^+2^ - ATPase (SERCA) inactivation (Fu et al. Nature, 2011, 473:528–31; Park et al. Proc. Natl. Acad. Sci. U.S.A., 2010, 107:19320-5). The increase in cytoplasmic calcium, in turn, leads to hyperactivation of the calcium-sensitive kinase, calcium/calmodulin dependent protein kinase II (CaMKII) (Ozcan et al. Cell Metab., 2012,15:739-51). Activated CaMKII induces hepatic glucose production through a pathway involving p38ɑ-mediated phosphorylation and nuclear translocation of a transcription factor called, FoxO1. Interestingly, a recent study reported that UNC-43, which is the C. elegans ortholog of CaMKII, phosphorylates the FoxO homologue DAF-16 and promotes its nuclear localization and transcriptional activity (Tao et al. Elife, 2013, 2:e00518).

Defective hepatic insulin signaling is another hallmark of T2D. Recent evidence has shown that liver in obese animals and humans is characterized by increased ER stress, which contributes to perturbed insulin signaling and insulin resistance. Remarkably, the same upstream CaMKII-p38ɑ pathway described above gives rise to another branch that activates the PERK branch of the ER stress pathway [1]. PERK activation leads to induction of the Akt inhibitor, Trb3, which suppresses insulin receptor signaling. As such, when the hepatic CaMKII–p38ɑ pathway is inhibited by genetic or pharmacologic means in obese mice, there is a marked improvement in metabolism, including lowering of blood glucose and insulin, and this occurs without any change in body weight, adiposity, food intake, or plasma glucagon. Additionally, in line with an improvement in hyperinsulinemia, liver-CaMKII- or liver-p38ɑ-inhibited mice are protected from fatty liver formation and exhibit lower plasma triglyceride levels. Relevance to humans is suggested by our recent survey of ~40 liver biopsy specimens from humans with varying body mass indices (BMI), which showed a correlation between molecular markers of the CaMKII-p38 pathway and increasing BMI. Moreover, primary human hepatocytes show all features of this pathway. These collective data suggest that inhibition of this pathway could provide the basis for a novel therapeutic approach to obesity-associated T2D.

Diabetes has been associated with structural and functional changes in the myocardium and is a risk factor for cardiac dysfunction. Additionally, certain diabetes drugs have been associated with increased risk for heart failure. In this regard, a key player in the pathogenesis of failing heart is hyperactivated CaMKII, which may be amplified in diabetes through hyperglycemia-induced *O*-GlcNAcylation of CaMKII (Erickson et al. Nature, 2013, 502:372-6). Sustained and excessive activation of CaMKII in cardiomyocytes leads to arrhythmias, maladaptive cardiac remodeling, and heart failure. Most importantly, CaMKII inhibition improves myocardial function, relieves heart failure, and lessens adverse remodeling after myocardial infarction in preclinical models (Anderson et al. J. Mol. Cell Cardiol., 2011, 51:468-73). Finally, we have shown that CaMKII hyperactivation promotes macrophage apoptosis, which is a major process in the progression of advanced atherosclerosis (Timmins et al. J. Clin. Invest., 2009, 119:2925-41).

The concept of cardiometabolic disease emphasizes the need to integrate the cellular pathophysiology of metabolic dysfunction and heart disease in T2D rather than considering them as separate entities. Moreover, the U.S. Food and Drug Administration (FDA) stated that all new diabetes drugs must be carefully assessed for long-term cardiovascular safety. Thus, the discovery of upstream pathways in different cells types that contribute to multiple pathological cardiometabolic processes in T2D may lead to a unique class of T2D therapies that have increased efficacy and safety than those that are currently available. The role of CaMKII hyperactivation in excessive hepatic glucose production and defective insulin signaling in the setting of obesity and in heart failure and atherosclerosis may represent an example of a common upstream pathway whose targeting may hold promise for a new therapeutic approach to cardiometabolic disease. Disclosure: Drs. Ozcan and Tabas are part of a company to investigate the potential use of inhibitors of the CaMKII-p38 pathway for T2D.
